# Editorial: Small non-coding RNAs in diseases

**DOI:** 10.3389/fmolb.2022.1086768

**Published:** 2023-01-04

**Authors:** Ke Zhang, Yong Sun Lee, Inhan Lee, Xiaoyong Bao

**Affiliations:** ^1^ Department of Pediatrics, University of Texas Medical Branch, Galveston, TX, United States; ^2^ Department of Cancer Biomedical Science, Graduate School of Cancer Science and Policy, National Cancer Center, Goyang-si, South Korea; ^3^ miRcore, Ann Arbor, MI, United States; ^4^ Institute for Translational Science, University of Texas Medical Branch, Galveston, TX, United States; ^5^ Institute for Human Infections and Immunity, University of Texas Medical Branch, Galveston, TX, United States

**Keywords:** small non-coding RNA, viral infections, RNA sequencing, inflammatory diseases, tRNA-derived RNA fragment

## Small non-coding RNAs in diseases

Non-coding RNAs (ncRNAs) are RNA molecules that are not translated into proteins. They show great potential to serve as novel biomarkers and prophylactic/therapeutic targets of human diseases, including cancer, neurodegenerative disease, viral infection, and inflammatory diseases (Skalsky and Cullen, 2010; Carpenter et al., 2013; Maoz et al., 2017; Damas et al., 2019; Wang et al., 2019). Based on their size, ncRNAs are generally grouped into long ncRNAs (lncRNAs, >200 nt) and small ncRNAs (sncRNAs, <200 nt). Major sncRNA classes include microRNAs (miRNAs), small nucleolar RNAs (snoRNAs), small nuclear RNAs (snRNAs), piwi-interacting RNA (piRNA), and tRNA-derived RNA Fragment (tRFs) (Liao et al., 2010; Fu et al., 2015; Hombach and Kretz, 2016; Haack et al., 2019). This special issue focused on the sncRNAs and their essential roles in physiological and pathological conditions to explore the mechanisms of sncRNA-mediated human diseases.

## miRNAs-regulated gastrointestinal inflammation

In addition to the roles of lncRNAs and circular RNAs in human inflammatory bowel diseases (Lin et al., 2020), miRNAs were also reported to regulate intestinal epithelial integrity in mice recently (Wang et al., 2017). In this Research Topic, an original research paper from Dr. Haque’s group found increased expression of miR-122 and miR-21 in stool in children with increased intestinal permeability (IIP) (Rashid et al.). The finding was from a large sample (n = 442) and through integrative analysis of miRNA profiling, mucosal inflammation, and intestinal permeability. The association of enhanced miR-122 and miR-21 expression with international permeability suggests that assessing these miRNAs may serve as a new diagnosis method of IIP in children who suffer diseases associated with intestinal barrier dysfunction.

This topic also reviewed miRNA-regulated primary biliary cholangitis (PBC) (Zhang et al.), with highlighted functions of miRNAs participating in PBC inflammation and related pathogenesis pathways, supporting ncRNAs being potential diagnostic biomarkers and/or therapeutic targets.

## sncRNAs and infectious diseases

This Research Topic includes three original research manuscripts about ncRNAs in viral infections. Two are related to severe acute respiratory syndrome coronavirus 2 (SARS-CoV-2) infection with high volumes of views (Zhang et al.; Wu et al.). The results from Dr. Bao’s group demonstrated that tRFs are the most impacted sncRNAs in the nasopharyngeal swab specimens of SARS-CoV-2-positive patients and SARS-CoV-2-infected airway epithelial cells. The group also revealed several SARS-CoV-2-derived sncRNAs with a predicted structure similar to tRFs (Wu et al.). The study also established physiologically relevant cell models for tRF functional studies and a new sequencing (seq) method, called T4 PNK (polynucleotide kinase)-RNA-seq, to accurately quantify the tRFs. These tools will benefit future sncRNA studies. Changes in a tRF profile have been demonstrated in other viral infections, with some being virus-specific and functionally crucial in viral replication (Wang et al., 2013; Ruggero et al., 2014; Selitsky et al., 2015). Therefore, rather than simply serving as biomarkers, any mechanisms associated with tRF biogenesis and function would also reveal potential targets to control viral replication. In addition to tRFs, four SARS-CoV-2-impacted lncRNAs were discovered by applying microarrays to peripheral blood mononuclear cells from healthy donors and COVID-19 patients (Zhang et al.). Although the lncRNA research is a bit off the topic scope, the urgent need to configure disease mechanisms of SARS-CoV-2 and the finding on the correlation between lncRNA expression in T cells and monocytes and the disease severity made us include this into the topic.

Respiratory syncytial virus (RSV) is the leading cause of acute lower respiratory tract infections in children worldwide. For the first time, Dr. Casola’s group showed a time-dependent increase in piRNAs in RSV-infected small airway epithelial (SAE) cells (Corsello et al.). Her group also identified genes related to cytoskeletal or Golgi organization and nucleic acid/nucleotide binding to be the most significantly altered by RSV-induced piRNAs, increasing the knowledge of the piRNA in viral infection and the potential of novel therapeutic targets for viral-mediated lung diseases.

We also included an original manuscript studying the roles of nematode miRNAs in T cell differentiation and cytokine production in macrophages (Soichot et al.), providing a new mechanism on how parasite miRNAs shape host gene expression.

## miRNA and intervertebral disc degeneration (IDD)

IDD is one of the main causes of lower back pain, but its pathogenesis mechanism remains unclear. An original research manuscript from Dr. Zhang’s group highlighted the importance of let-7b-5p plays a critical role in the maintenance of intervertebral disc structure and function (Zhuang et al.). The group showed that let-7b-5p in the exosome, secreted by nucleus pulposus stem cells derived from the degenerative intervertebral disc, can exacerbate annulus firbosus cell degeneration *via* inhibiting IGF1R expression and subsequently blocking the activation of the PI3K-Akt pathway.

## sncRNAs and their future directions

A review from Dr. Dutta’s group reviewed the functions and therapeutic implications of tRNA-derived small RNAs (Wilson and Dutta), which were found to be a dominant sncRNA group using Thermostable Group II Intron Reverse Transcriptase (TGIRT)-seq in bladder cancer samples (Su et al.). Their biogenesis mechanisms and functions in posttranscriptional gene silencing, regulating nascent RNA expression, altering protein translation, and affecting protein function were comprehensively discussed (Wilson and Dutta). The group also discussed the challenge when using tRFs as therapeutic targets, which is applicable to all sncRNA-based therapeutic development. Among them, the effectiveness of oligonucleotide delivery is a major issue. To overcome this, several tissue-specific delivery methods, including using lipid nanoparticles, N-acetylgalactosamine conjugation, and encapsulation of oligonucleotide in extracellular vesicles, were recently developed and showed the promise. In addition to the delivery issue, the oligonucleotide stability in circulation is another concern. tRFs/tRNAs are full of modifications, which may be functionally important including stabilizing sncRNAs. How to determine the modification location and their functions is challenging and raising difficulties of therapeutic oligo design. Overall, sncRNA-based therapeutic development is a challenging task. However, with more accumulating data revealing their biogenesis and function mechanisms, we may have the chance to target the pathways involved in biogenesis and functions, not necessarily direct oligo-based therapy, to prevent and treat the diseases.
